# High Altitude Pulmonary Edema

**DOI:** 10.21980/J8C35X

**Published:** 2020-04-15

**Authors:** Aubri Charnigo, Jennifer Yee

**Affiliations:** * The Ohio State University, Department of Emergency Medicine, Columbus OH

## Abstract

**Audience:**

This simulation was developed to educate emergency medicine residents on the diagnosis and management of high-altitude pulmonary edema (HAPE). This case is also appropriate for senior medical students and advanced practice providers. The principles of crisis resource management, teamwork, and communication are incorporated into the case.

**Introduction:**

High altitude pulmonary edema may present similarly to pneumonia with nonspecific symptoms, including decreased exercise tolerance, cough, dyspnea with exertion, and fever. Symptoms more specific to HAPE include dyspnea at rest, tachypnea, history of rapid ascent to high altitude, a lowlander patient exposed to high-altitude, or a highlander patient on re-entrance to high altitude after lowland stay. Laboratory and imaging workup may include infiltrates on chest x-ray and leukocytosis.^1^,^2^ Of the various forms of altitude sickness, HAPE has the highest fatality rate, estimated at 50% in travellers to the Himalayas who are unable to descend.^1^ Providers should inquire as to the current elevation of their facility, the patient’s recent altitude gain, and the rate of ascent. Treatment priorities include oxygen and immediate descent, as well as supplemental treatment with nifedipine and phosphodiesterase (PDE) inhibitors such as sildenafil or tadalafil.

**Educational Objectives:**

At the conclusion of the simulation session, learners will be able to:

1. obtain a thorough history relevant to altitude illnesses
2. develop a differential for dyspnea in a patient with environmental exposures
3. discuss prophylaxis and management of HAPE
4. discuss appropriate disposition of the patient including descent and subsequent appropriate level of care

**Educational Methods:**

This session was conducted using high-fidelity simulation followed by a debriefing session and lecture on the diagnosis and management of HAPE. Debriefing methods may be left to the discretion of participants, but the authors have utilized advocacy-inquiry techniques. This technique includes an observation statement, a statement describing the framework of the observer, and an invitation to review further to explore the participants’ frames. An example of this advocacy-inquiry is as follows: “I heard Sam suggest to the team that acetazolamide be given, but then I didn’t hear any follow-up discussion. Acetazolamide is often utilized for acute mountain illness prophylaxis or treatment. I wasn’t sure if the team did not hear his suggestion or disagreed with the treatment plan. Tell me more.” This scenario was designed as a simulation, but it could be adapted as an oral boards case.

**Research Methods:**

Our residents were provided with a survey at the completion of the simulation and debriefing to rate different aspects of the simulation, as well as to provide qualitative feedback.

**Results:**

Participants expressed positive feedback, with comments focused on appreciating the review of the presentation, diagnosis, and treatment of altitude illness. The emergency medicine residents surveyed currently practice in a low altitude setting and were appreciative to simulate a scenario to which they might otherwise not get exposure during their residency. Our simulation center’s feedback form is based on the Center of Medical Simulation’s Debriefing Assessment for Simulation in Healthcare (DASH) Student Version Short Form. This session received all 6 or 7 scores (consistently effective/very good or extremely effective/outstanding) other than a 5 for setting the stage, a 4 for maintaining an engaging context for learning, and two 5s for structured debriefing.

**Discussion:**

This was an effective method to review high altitude illness with learners that may otherwise get limited exposure to such clinical cases during residency. Learners had a broad range of differential diagnoses and demonstrated variable levels of knowledge related to the diagnosis and treatment of high-altitude illness. We used visual stimuli and a reminder from our nurse to reinforce for the learners that the case was taking place at a critical access emergency room at 11,000 feet of elevation.

**Topics:**

Medical simulation, high altitude pulmonary edema, high altitude cerebral edema, altitude sickness, emergency medicine, wilderness medicine.

## USER GUIDE

[Table t1-jetem-5-2-s78]

**Table t1-jetem-5-2-s78:** 

List of Resources:	
Abstract	78
User Guide	80
Instructor Materials	82
Operator Materials	92
Debriefing and Evaluation Pearls	96
Simulation Assessment	99

**Learner Audience:**

Medical students, interns, junior residents, senior residents

**Time Required for Implementation:**

Instructor Preparation: 30 minutes

Time for case: 20 minutes

Time for debriefing: 40 minutes

**Recommended Number of Learners per Instructor:**

4

**Topics:**

Medical simulation, high altitude pulmonary edema, high altitude cerebral edema, altitude sickness, emergency medicine, wilderness medicine.

**Objectives:**

By the end of this simulation session, the learner will be able to:

1. Obtain a thorough history, including current altitude, rate of ascent, peak altitude, patient’s baseline altitude, and assessment for other causes of dyspnea and altered mental status
2. Develop a differential for dyspnea in a patient with significant environmental exposures
3. Discuss prophylaxis and management of HAPE
4. Discuss appropriate disposition of the patient in both the field and in the high-altitude ED or clinic, including descent and subsequent appropriate level of care

### Linked objectives and methods

High altitude pulmonary edema is an uncommon ED presentation, and many of the symptoms are consistent with much more common diagnoses such as cardiogenic pulmonary edema or pneumonia. This scenario enforces the importance of obtaining a thorough history including ascent profile, baseline altitude, and maintaining a high clinical suspicion for altitude illness in the appropriate setting (objective 1). Learners will be required to develop a differential for dyspnea in a patient with environmental exposures (objective 2). Learners will need to identify the appropriate management of HAPE, as well as prophylaxis (objective 3), including appropriate disposition, including descent to lower altitude and inpatient level of care (objective 4). This simulation scenario allows learners to reinforce their HAPE management skills in a psychologically safe learning environment, and then receive formative feedback on their performance.

### Recommended pre-reading for instructor

We recommend that instructors review the Wilderness Medical Society Practice Guidelines for the Prevention and Treatment of Acute Altitude Illness: 2019 Update.^7^ Other suggested readings include materials listed below under the “References/suggestions for further reading” section.

### Results and tips for successful implementation

This simulation was written to be performed as a high-fidelity simulation scenario, but also may be used as a mock oral board case.

The case was written for emergency medicine residents and may be presented in an outdoor setting or simulation lab. We conducted this simulation with emergency medicine residents during the 2019–2020 academic year. Despite having a sign on the door that informed participants that they were moonlighting at an ED in the Rocky Mountains for this scenario, some groups needed further reinforcement to remind them that they are not in their usual ED location. Once participants realized they were at a high-altitude location, their differentials then immediately expanded to include altitude-related illnesses. For this reason, we did not have nursing consistently remind them that they were in a high-altitude critical access ED in order to minimize diagnostic anchoring.

During debriefing, participants expressed overall positive feedback including reviewing the spectrum of altitude related illnesses with approaches for both prophylaxis and treatment. The emergency medicine residents currently practice in a lowaltitude setting and appreciated the simulation of a scenario to which they might not otherwise be exposed in their clinical residency training.

Our simulation center’s feedback form is based on the Center of Medical Simulation’s Debriefing Assessment for Simulation in Healthcare (DASH) Student Version Short Form with the inclusion of required qualitative feedback if an element was scored less than a 6 or 7. This session received all 6 or 7 scores (consistently effective/very good or extremely effective/outstanding) other than a 5 for setting the stage, a 4 for maintaining an engaging context for learning, and two 5s for structured debriefing. Our form also includes an area for general feedback about the case at the end. Qualitative comments for the “setting the stage” category included, “Would have preferred more info from nurse about physically where we are and what type of hospital and what information we had?” reflecting the earlier sentiment that many residents needed further prompting that they were at a high-altitude ED instead of their typical practice location. The qualitative comment for the 4 score for the maintaining an engaging context for learning was, “When we were struggling in simulation it would have been nice to have a nudge or a clue rather than just more silence.” Our typical practice is to allow the participants to work through their differentials and give them opportunity to revisit their decision-making processes without prompting unless they are unable to move forward. All other scores in this area were 6s and 7s. Qualitative comments for debriefing were for both 5 scores and included, “Went well overall, I just didn’t know a whole lot about the topic,” and “great case,” so the authors wonder if one of those 5 scores was more reflective of the learner’s baseline knowledge about HAPE, rather than how the debriefing was structured or conducted.

## Supplementary Information



## Figures and Tables

**Table t2-jetem-5-2-s78:** 

#1	Complete blood count (CBC)
#2	Basic metabolic panel (BMP)
#3	Arterial blood gas (ABG)
#4	Troponin
#5	BNP
#6	D-dimer
#7	Urinalysis
#8	hCG qualitative
#9	Electrocardiogram (ECG)
#10	Chest Radiograph (CXR)
#11	Computed tomography pulmonary embolism (CT PE) study

**Table t3-jetem-5-2-s78:** 

**Vitals:**	Heart Rate (HR) 120	Blood Pressure (BP) 110/76	Respiratory Rate (RR) 36
	Temperature (T) 100.9°F	Oxygen Saturation (O_2_Sat) 73% on room air	
	Weight (Wt) 60 kg		

**Table t4-jetem-5-2-s78:** 

*Complete blood count (CBC)*	
White blood count (WBC)	16.0 ×1000/mm^3^
Hemoglobin (Hgb)	14.5 g/dL
Hematocrit (HCT)	45.0%
Platelet (Plt)	270 ×1000/mm^3^

**Table t5-jetem-5-2-s78:** 

*Basic metabolic panel (BMP)*	
Sodium	135 mEq/L
Chloride	100 mEq/L
Potassium	4.2 mEq/L
Bicarbonate (HCO_3_)	23 mEq/L
Blood Urea Nitrogen (BUN)	19 mg/dL
Creatine (Cr)	1.0 mg/dL
Glucose	85 mg/dL
Calcium	8.6 mg/dL

**Table t6-jetem-5-2-s78:** 

*Arterial blood gas (ABG)*	
pH	7.55
pCO2	15 mmHg
pO2	39 mmHg
HCO3	22 mEq/L
O2 saturation	73% on room air
Lactic acid	1.2 mmol/L

**Table t7-jetem-5-2-s78:** 

*Troponin*	<0.015 ng/mL

**Table t8-jetem-5-2-s78:** 

*NT-pro BNP*	110 pg/mL

**Table t9-jetem-5-2-s78:** 

*D-dimer*	<0.1 ug/mL

**Table t10-jetem-5-2-s78:** 

*Urinalysis (UA)*	
Leukocyte esterase	negative
Nitrites	negative
Blood	none
Protein	none
Ketones	none
Glucose	none
Color	dark yellow
White blood cells (WBC)	0–5 WBCs/high powered field (HPF)
Red blood cells (RBC)	0–5 RBCs/HPF
Squamous epithelial cells	0–5 cells/HPF
Specific gravity	1.015

**Table t11-jetem-5-2-s78:** 

*hCG qualitative*	negative

**Figure f1-jetem-5-2-s78:**
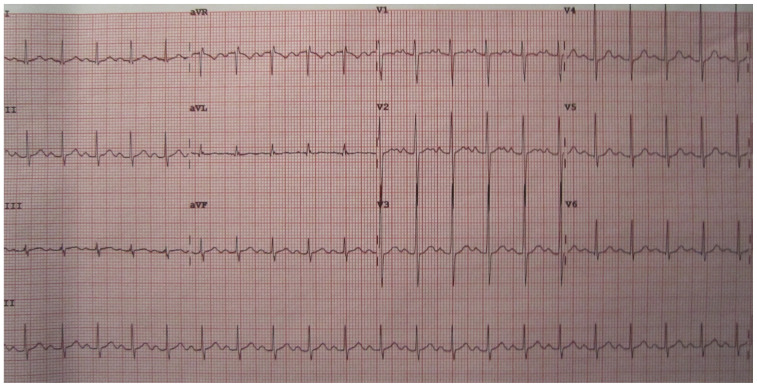


**Figure f2-jetem-5-2-s78:**
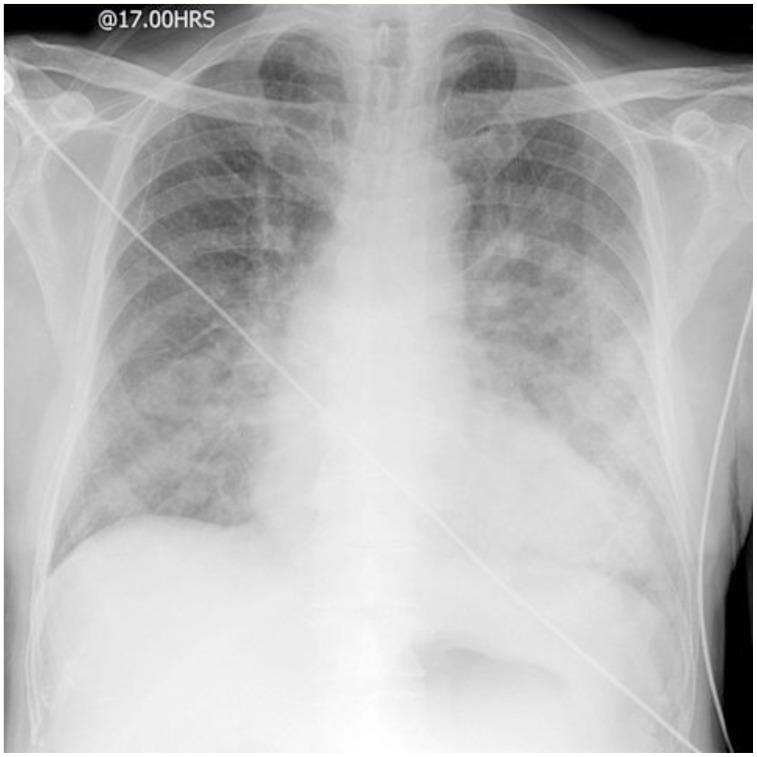


**Figure f3-jetem-5-2-s78:**
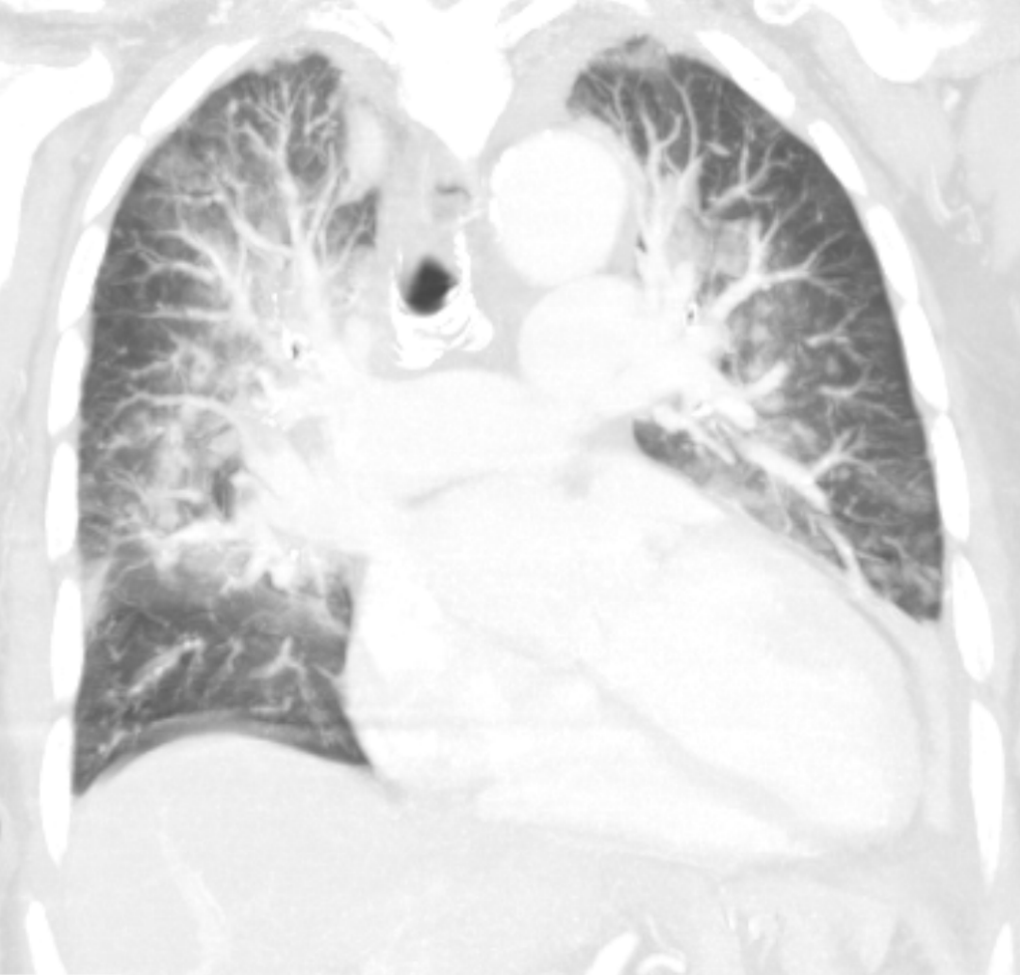


**Table t12-jetem-5-2-s78:** SIMULATION EVENTS TABLE:

Minute (state)	Participant action/trigger	Patient status (simulator response) & operator prompts	Monitor display (vital signs)
0:00 (Baseline)	Patient moved into bed in the emergency department.	Participants should begin by placing the patient on a monitor, obtaining a history and performing a physical exam.	No vitals should be displayed until requested T 100.9 HR 120 BP 110/76 RR 36 O2 73% RA
04:00	IV placed, labs drawn. Participant should apply supplemental oxygen, perform a thorough physical exam, obtain ECG, order labs and CXR.	If the team administers oxygen, tachypnea and O2 saturation will improve. If the team does not administer oxygen, patient will become obtunded, further desaturate and will begin to have a drop in respiratory rate. If team intubates successfully on first attempt, hypoxia will improve to 92%. If team has difficulty intubating (more than 2 attempts or a prolonged attempt), go straight to PEA arrest (15:00 vitals).	On a non-rebreathing mask: T 100.9 F HR 110 BP 110/76 RR 35 O2 86% on 15L O2 On a positive pressure mask: T 100.9 F HR 110 BP 110/76 RR 30 O2 90% on BiPAP or CPAP Intubated: T 100.9F HR 90 RR (set by team) - bagged O2 92% bag valve mask (BVM) Without oxygen: T 100.9F HR 130 BP 90/60 RR 26 O2 65% on RA
10:00	Administer nifedipine and oxygen.	If team administers nifedipine to the non-intubated patient, RR decreases to 26 and oxygenation improves to 92%. It not, vitals remain unchanged from previous step. If team administers nifedipine to the non-intubated patient, oxygenation improves to 94%. It not, vitals remain unchanged from previous step. If oxygen is still not administered regardless if nifedipine is given, patient’s respiratory rate will slow as the patient decompensates (from 8:00–10:00). After two more minutes (10:00), the patient will go into PEA cardiac arrest secondary to respiratory arrest for two rounds of appropriately performed ACLS until ROSC is achieved.	Nifedipine given with oxygen: T 100.9 F HR 100 BP 110/76 RR 24 O2 92% on 15L O2 or positive pressure oxygen Intubated with nifedipine: T 100.9F HR 90 RR (set by team) - bagged O2 94% BVM Without oxygen: T 100.9F HR 70 BP 90/60 RR 16 O2 60% on RA
15:00 (A) PEA arrest/ROSC	If patient arrests, ROSC will be achieved with 2 rounds of appropriate ACLS, as long as appropriate bag-valve mask respirations or airway secured. OSH hospital at lower elevation should be contacted for direct admission to ICU and transport arranged.	Once ICU is contacted, they will ask why the patient arrested. They may provide further treatment guidance as felt appropriate per facilitator. When the transfer center is contacted, they should offer admission to an ICU several minutes away at the same altitude or a hospital that is an hour away by ground and is 3000 feet lower in elevation. If the nearby hospital is chosen, the transfer center will call back in 3 minutes and will state they are currently on diversion.	During arrest: T 100.9F HR 70 (PEA) RR (set by team) - bagged O2 86% BVM Post arrest: T 100.9F HR 90 RR (set by team) - bagged O2 92% BVM
15:00 (B) ICU should be called for admission	Team contacts OSH at lower elevation for ICU admission and arranges for transport.	Case ends. When the transfer center is contacted, they should offer admission to an ICU several minutes away at the same altitude or a hospital that is an hour away by ground and is 3000 feet lower in elevation. If the nearby hospital is chosen, the transfer center will call back in 3 minutes and will state they are currently on diversion.	Oxygen given, no nifedipine given: T 100.9 F HR 110 BP 110/76 RR 30 O2 86% on 15L O2 (90% on BiPAP/CPAP Oxygen and Nifedipine given: T 100.9 F HR 100 BP 110/76 RR 26 O2 92% on 15L O2 (also 92% on BiPAP/CPAP) Intubated with nifedipine: T 100.9F HR 90 RR (set by team) - bagged O2 94% BVM Intubated without nifedipine: T 100.9F HR 90 RR (set by team) - bagged O2 92% BVM

**Table t13-jetem-5-2-s78:** 

**Critical Actions:** Place patient on monitor and obtain a full set of vitals, including temperature and pulse oximetry Apply supplemental oxygen Obtain IV access Obtain further history, including current altitude and patient’s rate of ascent Obtain an ABG, labs, CXR, and ECG Identify pulmonary edema on CXR Treat with nifedipine or an PDE-5 inhibitor Cover for possible pneumonia with antibiotic therapy Arrange for transport to a hospital at lower elevation with critical care capabilities	0:00

**Table t14-jetem-5-2-s78:** 

	Milestone	Did not achieve level 1	Level 1	Level 2	Level 3
1	**Emergency Stabilization (PC1)**	□ Did not achieve Level 1	□ Recognizes abnormal vital signs	□ Recognizes an unstable patient, requiring intervention Performs primary assessment Discerns data to formulate a diagnostic impression/plan	□ Manages and prioritizes critical actions in a critically ill patient Reassesses after implementing a stabilizing intervention
2	**Performance of focused history and physical (PC2)**	□ Did not achieve Level 1	□ Performs a reliable, comprehensive history and physical exam	□ Performs and communicates a focused history and physical exam based on chief complaint and urgent issues	□ Prioritizes essential components of history and physical exam given dynamic circumstances
3	**Diagnostic studies (PC3)**	□ Did not achieve Level 1	□ Determines the necessity of diagnostic studies	□ Orders appropriate diagnostic studies. Performs appropriate bedside diagnostic studies/procedures	□ Prioritizes essential testing Interprets results of diagnostic studies Reviews risks, benefits, contraindications, and alternatives to a diagnostic study or procedure
4	**Diagnosis (PC4)**	□ Did not achieve Level 1	□ Considers a list of potential diagnoses	□ Considers an appropriate list of potential diagnosis May or may not make correct diagnosis	□ Makes the appropriate diagnosis Considers other potential diagnoses, avoiding premature closure
5	**Pharmacotherapy (PC5)**	□ Did not achieve Level 1	□ Asks patient for drug allergies	□ Selects an medication for therapeutic intervention, consider potential adverse effects	□ Selects the most appropriate medication and understands mechanism of action, effect, and potential side effects Considers and recognizes drug-drug interactions
6	**Observation and reassessment (PC6)**	□ Did not achieve Level 1	□ Reevaluates patient at least one time during case	□ Reevaluates patient after most therapeutic interventions	□ Consistently evaluates the effectiveness of therapies at appropriate intervals
7	**Disposition (PC7)**	□ Did not achieve Level 1	□ Appropriately selects whether to admit or discharge the patient	□ Appropriately selects whether to admit or discharge Involves the expertise of some of the appropriate specialists	□ Educates the patient appropriately about their disposition Assigns patient to an appropriate level of care (ICU/Tele/Floor) Involves expertise of all appropriate specialists
9	**General Approach to Procedures (PC9)**	□ Did not achieve Level 1	□ Identifies pertinent anatomy and physiology for a procedure Uses appropriate Universal Precautions	□ Obtains informed consent Knows indications, contraindications, anatomic landmarks, equipment, anesthetic and procedural technique, and potential complications for common ED procedures	□ Determines a back-up strategy if initial attempts are unsuccessful Correctly interprets results of diagnostic procedure
20	**Professional Values (PROF1)**	□ Did not achieve Level 1	□ Demonstrates caring, honest behavior	□ Exhibits compassion, respect, sensitivity and responsiveness	□ Develops alternative care plans when patients’ personal beliefs and decisions preclude standard care
22	**Patient centered communication (ICS1)**	□ Did not achieve level 1	□ Establishes rapport and demonstrates empathy to patient (and family) Listens effectively	□ Elicits patient’s reason for seeking health care	□ Manages patient expectations in a manner that minimizes potential for stress, conflict, and misunderstanding. Effectively communicates with vulnerable populations, (at risk patients and families)
23	**Team management (ICS2)**	□ Did not achieve level 1	□ Recognizes other members of the patient care team during case (nurse, techs)	□ Communicates pertinent information to other healthcare colleagues	□ Communicates a clear, succinct, and appropriate handoff with specialists and other colleagues Communicates effectively with ancillary staff

## INSTRUCTOR MATERIALS

**Case Title:** High Altitude Pulmonary Edema

**Case Description & Diagnosis (short synopsis):** Patient is a 27-year-old healthy female hiker who presents from the wilderness with dyspnea. Per EMS, the patient was backpacking in the Rocky Mountains with friends. Throughout the day, she was having increasing trouble keeping up and appeared to be mildly short of breath. The shortness of breath worsened and did not resolve with rest, despite more frequent breaks to rest and a slower pace. When she did not improve while resting in camp, her friends hiked down to town to call for rescue. She was evacuated in a litter by search and rescue. She is currently too dyspneic to speak. Participants should obtain an oxygen saturation, temperature, electrocardiogram (ECG), arterial blood gas (ABG), chest x-ray (CXR), and blood work. Diagnostics will reveal low oxygen saturation, low partial pressure of oxygen (PaO2), mild leukocytosis, and CXR findings consistent with pulmonary edema. The patient should be treated with oxygen and nifedipine with additional consideration of sildenafil or tadalafil treatment. Given the patient’s leukocytosis, infiltrates on CXR, and fever, it would be reasonable to concurrently treat for pneumonia with antibiotics. The patient should be transferred by ground to a hospital with ICU capabilities at lower elevation.

**Equipment or Props Needed:**

High fidelity simulation mannequin

Flannel shirt, jeans, and hiking boots to place on the mannequin to signify patient has been hiking

Angiocaths for peripheral intravenous access = 18g, 20g, 22g

Cardiac monitor

Pulse oximetry

IV pole

Normal saline (1L ×2)

Nasal cannula oxygen

Non-rebreather mask

Continuous positive airway pressure (CPAP) or Bilevel positive airway pressure (BiPAP) mask Simulated medications with labeling: nifedepine, acetazolamide, sildenafil, dexamethasone

**Confederates needed:**

primary nurse

**Stimulus Inventory:**

[Table t2-jetem-5-2-s78]

**Background and brief information:** Search and rescue were called by the patient’s friend for shortness of breath. They brought the patient to the ED after a ground evacuation from a backpacking camp 8 miles from the nearest paved road.

**Initial presentation:** The patient is a 27-year-old female with no past medical history who presents from the wilderness via EMS with severe dyspnea.

She reports shortness of breath and chest tightness. She is occasionally coughing. Her cough is productive of pink frothy sputum.

She is too dyspneic to converse in full sentences. She denies headache, ataxia or vomiting. She endorses nausea. Last meal was 12 hours ago. Her friends tried to feed her in camp, but she was too nauseous and dyspneic to eat. She hiked 8 miles today and drank 2 liters of water.

If asked, she lives in Ohio (sea level) and flew to Colorado 2 days ago for a backpacking trip with friends. She hiked from 8,000 feet of elevation to 12,000 feet of elevation today. She noticed some initial exercise intolerance that worsened as she continued to hike.

The nurse may share that the hospital is a critical access hospital at 11,000 ft. It has an emergency room and 25 medical beds. There is not an inpatient critical care unit.

- Past medical history: none
- Past surgical history: appendectomy at age 12
- Medications: combined estrogen/progesterone oral contraceptive pill (OCP)
- Allergies: none
- Family history: noncontributory.
- Weight: 60 kg

Assessment: Lying supine, extremely tachypneic with little ability to converse, appears to be cyanotic and somewhat drowsy, occasionally coughing

**How the scenario unfolds:** Patient is a 27-year-old healthy female hiker who presents from the wilderness with dyspnea. Per EMS, the patient was backpacking in the Rocky Mountains with friends. Throughout the day, she was having increasing trouble keeping up and appeared to be mildly short of breath. The shortness of breath worsened and did not resolve with rest, despite more frequent breaks to rest and a slower pace. When she did not improve while resting in camp, her friends hiked down into town to call for rescue. She was evacuated in a litter by a search and rescue team. She is currently too dyspneic to converse in full sentences. Participants should obtain an oxygen saturation (O2 sat), temperature, ECG, ABG, chest x-ray, and blood work. Diagnostics will reveal a respiratory alkalosis with low PaO2, hypoxia, an elevated WBC, and pulmonary edema. The patient should be treated with oxygen and nifedipine with consideration for use of sildenafil or tadalafil. Given the patient’s elevated WBC, infiltrates on CXR and fever, it would be reasonable to concurrently treat for pneumonia with antibiotics. Patient’s tachypnea, tachycardia, and hypoxia improve with oxygenation with a modest improvement with the addition of nifedipine. If providers do not apply supplemental oxygen, the patient will become progressively more somnolent and bradycardic until pulseless electrical activity (PEA) arrest. Return of spontaneous circulation (ROSC) will be achieved once patient is appropriately ventilated with a bag-valve mask or intubated. When the transfer center is contacted, they should offer admission to an ICU several minutes away at the same altitude or a hospital that is an hour away by ground and is 3000 feet lower in elevation. If the nearby hospital is chosen, the transfer center will call back in 3 minutes and will state they are currently on diversion. The patient should be evacuated by ground to a hospital with ICU capabilities at lower elevation.

**Critical actions:**

1. Place patient on monitor and obtain a full set of vitals, including temperature and pulse oximetry
2. Apply supplemental oxygen
3. Obtain IV access
4. Obtain further history, including current altitude and patient’s rate of ascent
5. Obtain an ABG, labs, CXR, and ECG
6. Identify pulmonary edema on CXR
7. Treat with nifedipine or a PDE-5 inhibitor
8. Cover for possible pneumonia with antibiotic therapy
9. Arrange for transport to a hospital at lower elevation with critical care capabilities

**Case Title:** High Altitude Pulmonary Edema

**Chief Complaint:** Patient is a 27-year-old female who presents from the wilderness by EMS for severe dyspnea

[Table t3-jetem-5-2-s78]

**General Appearance:** lying supine in bed, visibly short of breath with labored respirations, cyanotic, having difficulty conversing secondary to dyspnea, drowsy

**Primary Survey:**

- **Airway:** intact
- **Breathing:** diffuse rales, severely tachypneic, accessory muscle use present, perioral cyanosis
- **Circulation:** tachycardic rate and regular rhythm. 2+ symmetric pulses. Cyanosis present to distal fingers and to toes.

**History:**

- **History of present illness:** Patient is a 27-year-old female with no previous medical history (PMH) who presents from the wilderness via EMS with severe dyspnea and reported hallucinations without other signs of altered mental status.

She is too dyspneic to converse in full sentences. She denies headache, ataxia or vomiting. She endorses nausea. Last meal was 12 hours ago. Her friends tried to feed her in camp, but she was too nauseous to eat. She hiked 8 miles today and drank 2 L of water.

If asked, she lives in Ohio (sea level) and flew to Colorado 2 days ago for a backpacking trip with friends. She hiked from 8000 ft of elevation to 12,000 ft of elevation today.

The nurse may share that the hospital is a critical access hospital at 11,000 ft. It has an emergency room and 25 floor-level beds.

- **Past medical history**: none
- **Past surgical history:** appendectomy at age 12
- **Medications:** combined estrogen/progesterone OCP
- **Allergies:** none
- **Social history:** social drinker, had 2 beers last night. No other alcohol this week. Denies drug use. Not currently sexually active.
- **Family history:** noncontributory

**Secondary Survey/Physical Examination:**

- **General appearance:** Lying in bed, severely tachypneic with difficulty speaking, appears cyanotic, drowsy
- **HEENT:**
  ○ **Head:** within normal limits other than perioral cyanosis
  ○ **Eyes:** within normal limits
  ○ **Ears:** within normal limits
  ○ **Nose:** within normal limits
  ○ **Throat:** within normal limits
- **Neck:** within normal limits
- **Heart:** tachycardic, otherwise, within normal limits
- **Lungs:** diffuse rales in all lung fields, very tachypneic at rest, using accessory muscles to breathe
- **Abdominal/GI:** within normal limits
- **Genitourinary:** within normal limits
- **Rectal:** within normal limits
- **Extremities:** Cyanosis present to distal fingers and to toes.
- **Back:** within normal limits
- **Neuro:** Drowsy but awake and oriented ×3. No focal motor or sensory deficits. Coordinated in all extremities, if tested. No clonus. Cranial nerves intact. GCS 15 (E4V5M6).
- **Skin:** within normal limits other than cyanosis as noted above
- **Lymph:** within normal limits
- **Psych:** within normal limits

**Results:**

[Table t4-jetem-5-2-s78]

[Table t5-jetem-5-2-s78]

[Table t6-jetem-5-2-s78]

[Table t7-jetem-5-2-s78]

[Table t8-jetem-5-2-s78]

[Table t9-jetem-5-2-s78]

[Table t10-jetem-5-2-s78]

[Table t11-jetem-5-2-s78]

*Electrocardiogram (EKG)*

Heilman, J. Sinus tachycardia as seen on ECG. In: Wikimedia Commons. https://commons.wikimedia.org/wiki/File:Sinustachy.JPG. Published June 15, 2012. CC BY-SA 3.0.[Fig f1-jetem-5-2-s78]

*Chest Radiograph (CXR)*

Jones, Jeremy. AP portable CXR of a patient in acute pulmonary edema. In: Wikimedia Commons. https://commons.wikimedia.org/w/index.php?curid=7371914. Published July 23, 2009. CC BY-SA 3.0. ros.[Fig f2-jetem-5-2-s78]

*Computed Tomography Pulmonary Embolism Study*

Hellerhoff. Pulmonary edema in CT-scan coronal MPR. https://commons.wikimedia.org/wiki/File:18-01-Lungenoedem_CT_coronar.png. In: Wikimedia Commons. Published December 5, 2010. CC BY-SA 3.0. ros.[Fig f3-jetem-5-2-s78]

## OPERATOR MATERIALS

[Table t12-jetem-5-2-s78]

### Diagnosis

High Altitude Pulmonary Edema

### Disposition

Transfer to a hospital at lower elevation (descent required), then ICU

## DEBRIEFING AND EVALUATION PEARLS

### High Altitude Pulmonary Edema

#### Pearls

The spectrum of altitude sickness includes acute mountain sickness (AMS), high altitude cerebral edema (HACE) and high-altitude pulmonary edema (HAPE). HAPE is the least common manifestation of altitude sickness but has the highest fatality without descent.

The incidence of HAPE is less than 1% in the general mountaineering population of the Alps, but rises to 10% when non-susceptible individuals ascend to 4559 meters (m) in 24 hours and to 60% in susceptible individuals.^1^ In patients whose partial pressure of oxygen can be rapidly reduced to sea-level, there is generally rapid and complete recovery. However, the fatality rate was estimated at 50% in hikers in the Himalayas for whom descent was not possible and oxygen was not available.^1^

High altitude pulmonary edema is a clinical diagnosis; however, the Lake Louis Consensus Criteria are international guidelines for the diagnosis of HAPE for research purposes. The Lake Louis Criteria require at least 2 of the following symptoms: dyspnea at rest, cough, weakness or decreased exercise performance, chest tightness or congestion AND 2 of the following signs: crackles or wheezing in at least one lung field, central cyanosis, tachypnea, or tachycardia. Hemoptysis or pink-frothy sputum are later signs. Neurological symptoms may be secondary to hypoxic encephalopathy or a sign of concurrent HACE, which is present in up to 70% of cases. Patients may have a low-grade fever and universally have hypoxia and respiratory alkalosis. Radiologic studies are consistent with pulmonary edema.

High altitude pulmonary edema can be diagnosed based on history and physical, chest x-ray, and oxygen saturation alone, but blood work will show a respiratory alkalosis on arterial blood gas (ABG) and potentially a mild nonspecific leukocytosis.

The pathophysiology of HAPE is related to induced pulmonary hypertension secondary to hypoxemia-driven pulmonary vasoconstriction. Hypoxia occurs secondary to the decreased partial pressure of oxygen at high altitude. This pulmonary vasoconstriction causes an alveolar capillary leak, resulting in pulmonary edema. 5,6 Certain individuals are more susceptible to HAPE than others, independent of age, gender, or level of fitness. It is hypothesized that these individuals may have impaired pulmonary nitric oxide synthesis, resulting in an exaggerated pulmonary hypertensive response. Individuals susceptible to HAPE are also more likely to have impaired alveolar fluid reabsorption due to impaired epithelial sodium transport.

High altitude pulmonary edema may present similarly to pneumonia with cough, dyspnea, a low-grade fever and a mild leukocytosis. Pneumonia should remain in the differential, but a history of high-altitude exposure makes HAPE more likely.

The first-line treatment for HAPE is immediate descent of at least 1000 m in altitude, with minimal exertion on the part of the patient, if possible.^7^ In mild cases, this may be sufficient for relief of symptoms. Because exercise can exacerbate symptoms, the patient may need assistance in descent. Upon arrival to a hospital, the patient should be treated with supplemental oxygen. If oxygen is available prior to descent or descent is not possible, oxygen should be applied with goal SpO2 >90%.^7^ Oxygen and descent are the mainstays of treatment of HAPE. Pharmacologic treatments should be considered in patients who are unable to achieve an SpO2 >90% with oxygen alone or who are unable to immediately descend. Continuous positive airway pressure can be considered when available if the patient is unable to maintain oxygen saturation over 90% with supplemental oxygen alone.^7^

Nifedipine is currently the first-line pharmacologic treatment for HAPE (20 mg extended release every 8 hours or 30 mg extended release every 12 hours).^7^,^8^ Initial studies of PDE inhibitors sildenafil and tadalafil for the treatment and prevention of HAPE show good results with less systemic blood pressure reduction compared to nifedipine.^3^,^4^

When descent is not possible, administer oxygen and nifedipine, and consider a portable hyperbaric chamber (such as Gamow® or Certec® Bags).^7^,^8^ There is a risk of recurrence of symptoms on emergence from hyperbaric therapy.

Graded ascent and acclimatization are key to prevention of HAPE.^7^ There is no consensus on the recommended rate of ascent, but one source suggests that anyone with a history of HAPE should limit ascent to no more than 1155 ft (350 meters) a day.^2^ Other aspects of prevention include avoidance of alcohol, sleep aids, strenuous exercise, and low sleeping altitude. 2 Prophylaxis is only recommended for those with a history of HAPE. Nifedipine extended release 20 mg every 8 hours or 30 mg every 12 hours has been shown to be effective in these patients.^7^,^8^ Salmeterol 125 mcg inhaled twice daily has also been shown to be effective prophylaxis in HAPE-susceptible individuals.^8^ Studies on the efficacy of sildenafil and tadalafil for HAPE prevention are on-going, but initial results show they are superior to placebo.^3^,^4^ There is currently no evidence to support the use of dexamethasone in the treatment of HAPE; however, there is a high rate of concurrence of HACE with HAPE. If patients have encephalopathy, they may have hypoxia-induced encephalopathy versus concurrent HACE, and it would be reasonable to consider treating with dexamethasone.

Acute mountain sickness (AMS) is characterized by headache and at least one of the following: dizziness or lightheadedness, fatigue or weakness, nausea/vomiting/anorexia, insomnia (Lake Louis Criteria). Risk factors for AMS include history of AMS in the past, rapid or high ascent, and obesity. It is common and does not mandate descent unless symptoms progress to HACE or HAPE, but the patient should rest and/or slow the rate of ascent.

High altitude cerebral edema represents the progression of AMS to include the development of cerebral edema. It is defined by the symptoms of AMS with additional symptoms of ataxia, altered level of consciousness, and/or severe drowsiness. Symptoms can progress to coma. The first-line treatment of HACE is immediate descent. HACE can be treated with dexamethasone (8 mg once, then 4 mg every 6 hours),^7^ acetazolamide (as an adjunctive therapy, at the AMS treatment dose, below), supplemental oxygen and hyperbarics (if descent is not possible).

Acetazolamide is the gold standard for AMS prophylaxis. Acetazolamide 125 mg oral twice daily starting 1–2 days prior to ascent is well-studied prophylaxis for AMS/HACE.^7^ It does not have a role in prophylaxis for HAPE. Acetazolamide can also be used at a higher dose (250 mg oral twice daily) for the treatment of AMS.^7^

This case is designed for the learners to include consideration of descent as the patient’s disposition because this is a key take-away in the management of altitude illness. Because the patient is hypoxic to the 70’s and does not fully respond to oxygen therapy alone, ICU is the appropriate final disposition. However, in practice, these patients can rapidly improve with descent and oxygen to the extent that they can potentially be admitted to a medical-surgical level of care or even discharged home from the emergency room after several hours of treatment and monitoring (possibly with oxygen upon discharge) and clear instructions to rest and that they are not to re-ascend.

#### Other debriefing points

Closed-loop communication amongst team: was it used? Why or why not? Were there any implications of this during case execution?

## SIMULATION ASSESSMENT

*High Altitude Pulmonary Edema*

Learner: _________________________________________

***Assessment Timeline***

This timeline is to help observers assess their learners. It allows observer to make notes on when learners performed various tasks, which can help guide debriefing discussion.

[Table t13-jetem-5-2-s78]

**Critical Actions:**

□ Place patient on monitor and obtain full set of vitals, including temperature and O2 saturation
□ Apply supplemental oxygen
□ Obtain IV access
□ Obtain further history, including current altitude and patient’s rate of ascent
□ Check an ABG, labs, CXR, ECG
□ Identify pulmonary edema on CXR
□ Treat with nifedipine or PDE-5 inhibitor
□ Cover for possible pneumonia with antibiotic therapy
□ Arrange for transport to a hospital at lower elevation with critical care capabilities

**Summative and formative comments:**

**Milestones assessment:**

[Table t14-jetem-5-2-s78]
