# Supplementary material for: High Altitude Pulmonary Edema
Source: J Educ Teach Emerg Med. 2020 Apr 15;5(2):S78–S103. doi: 10.21980/J8C35X (PMC10332563; doi:10.21980/J8C35X)
Supplement: Supplementary file 1 [file jetem-5-2-s78.pptx]

## Slide 1
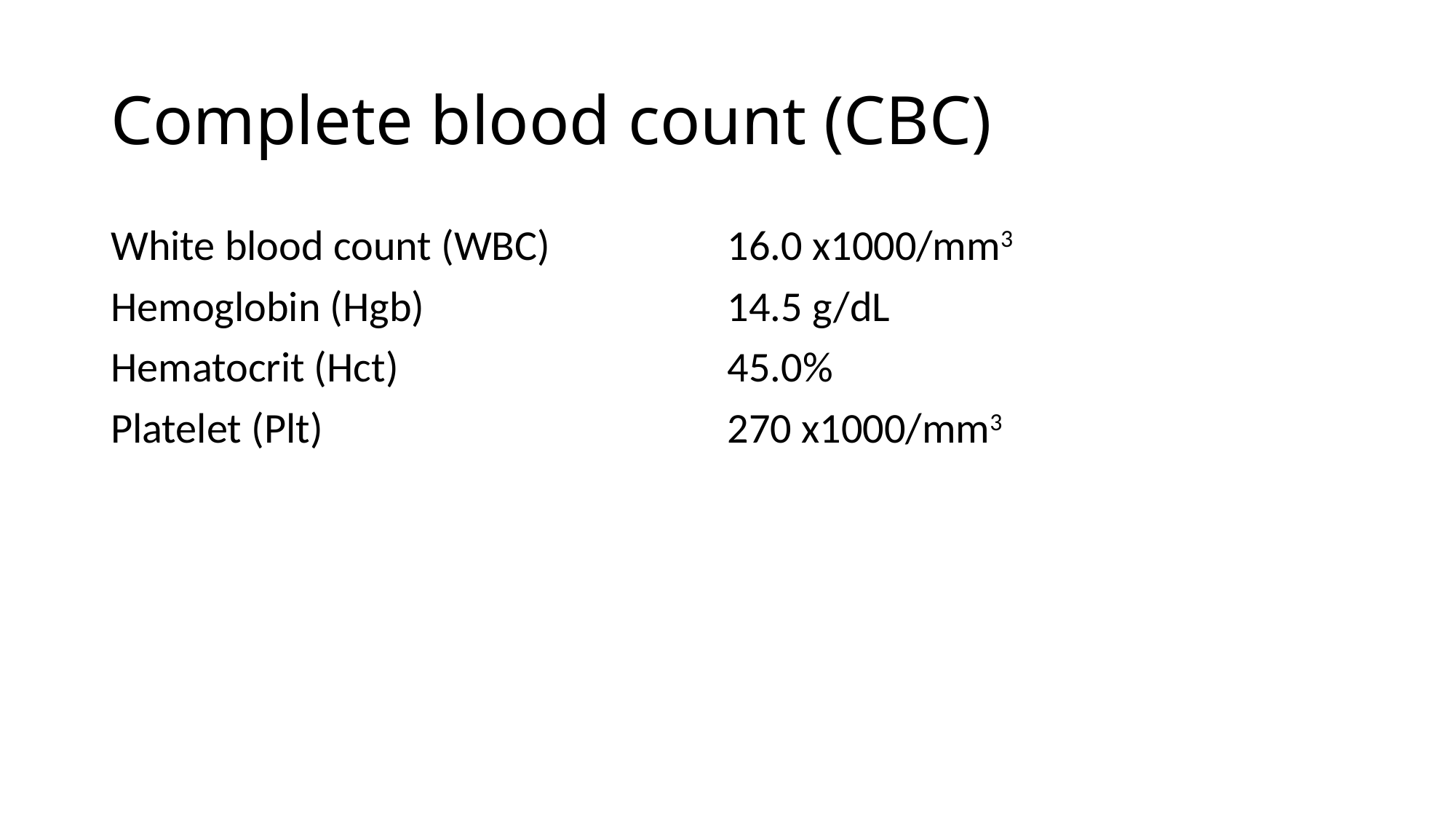

# Complete blood count (CBC)
White blood count (WBC)
Hemoglobin (Hgb)
Hematocrit (Hct)
Platelet (Plt)
16.0 x1000/mm3
14.5 g/dL
45.0%
270 x1000/mm3

## Slide 2
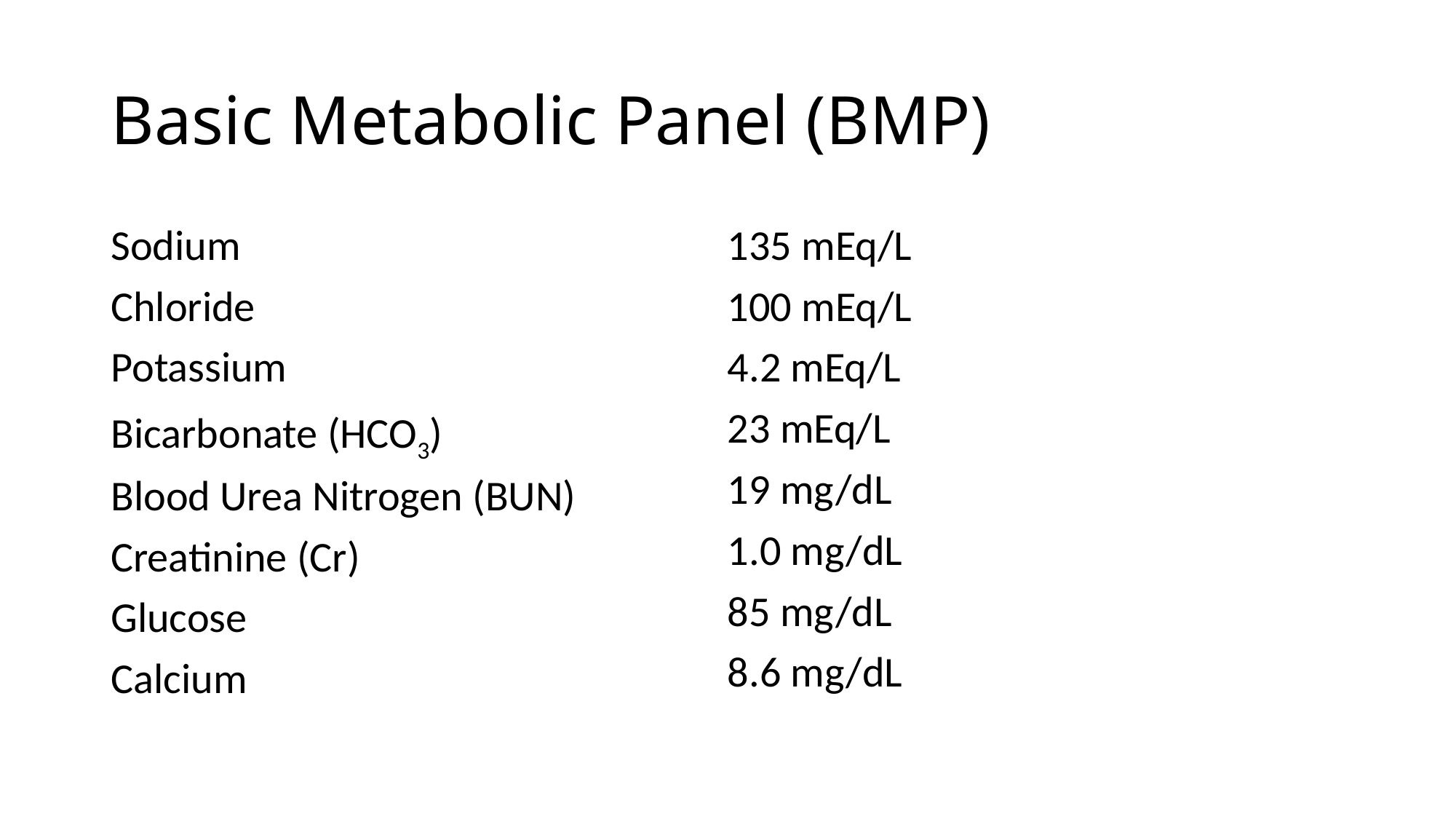

# Basic Metabolic Panel (BMP)
Sodium
Chloride
Potassium
Bicarbonate (HCO3)
Blood Urea Nitrogen (BUN)
Creatinine (Cr)
Glucose
Calcium
135 mEq/L
100 mEq/L
4.2 mEq/L
23 mEq/L
19 mg/dL
1.0 mg/dL
85 mg/dL
8.6 mg/dL

## Slide 3
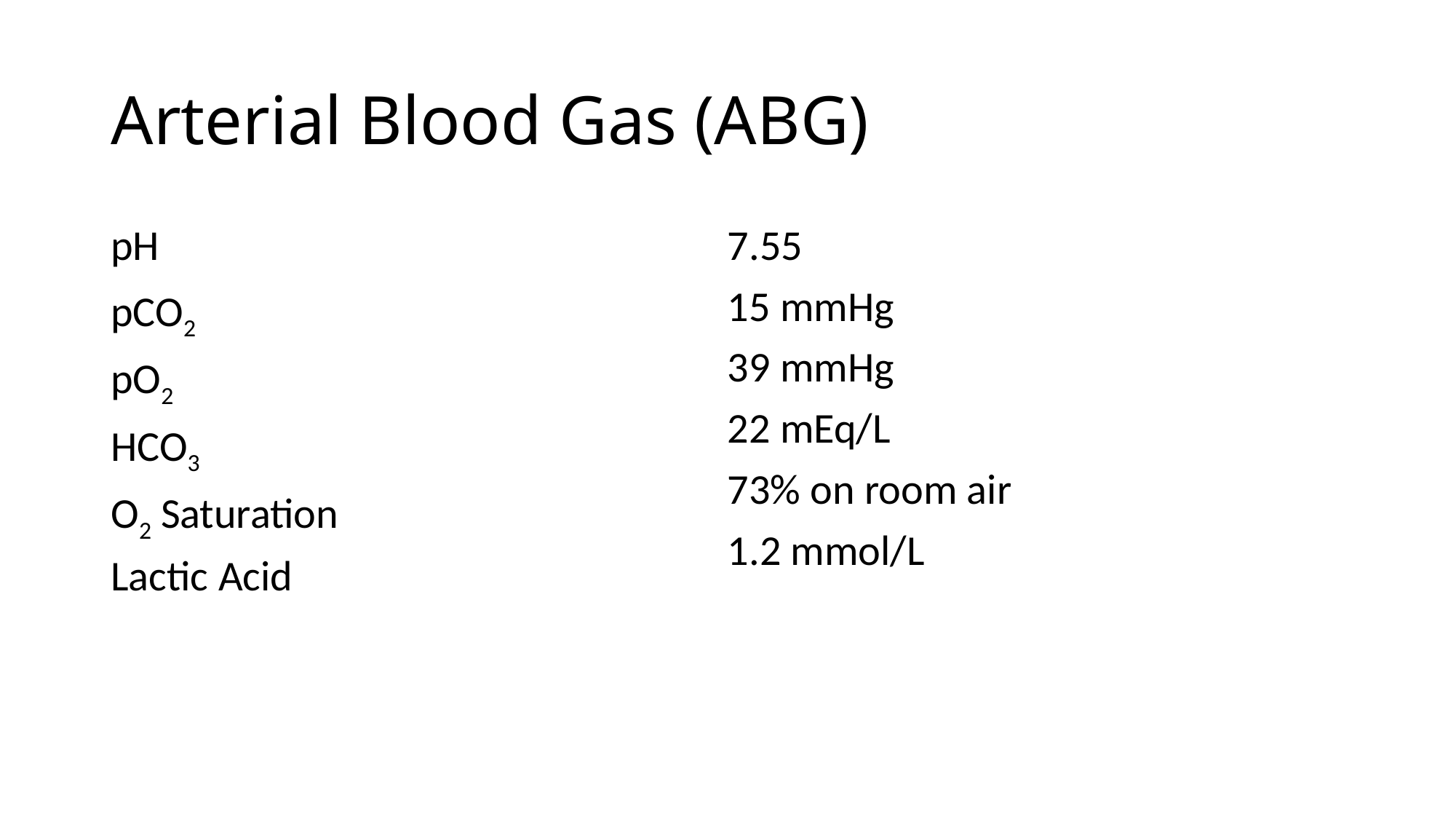

# Arterial Blood Gas (ABG)
pH
pCO2
pO2
HCO3
O2 Saturation
Lactic Acid
7.55
15 mmHg
39 mmHg
22 mEq/L
73% on room air
1.2 mmol/L

## Slide 4
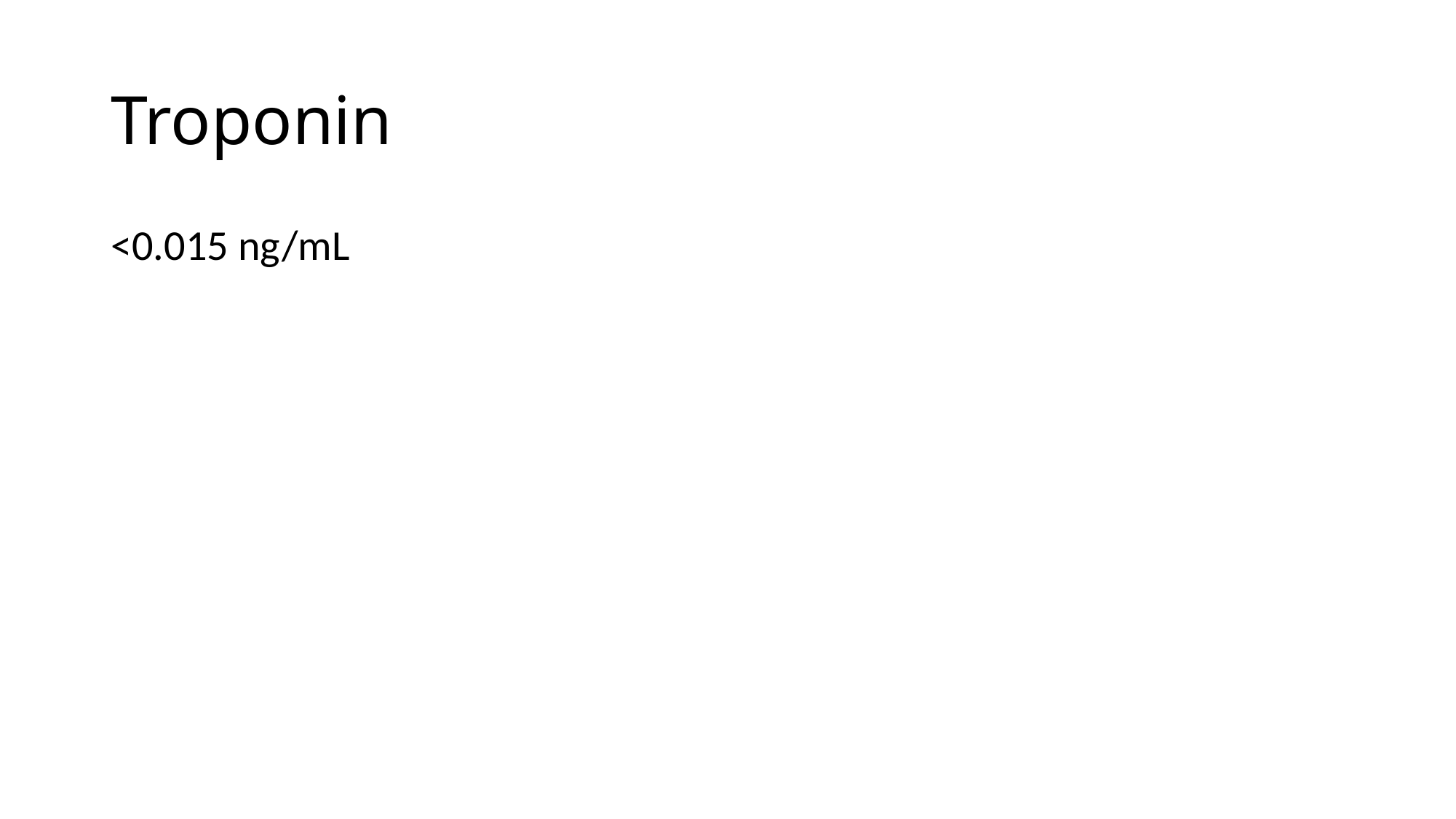

# Troponin
<0.015 ng/mL

## Slide 5
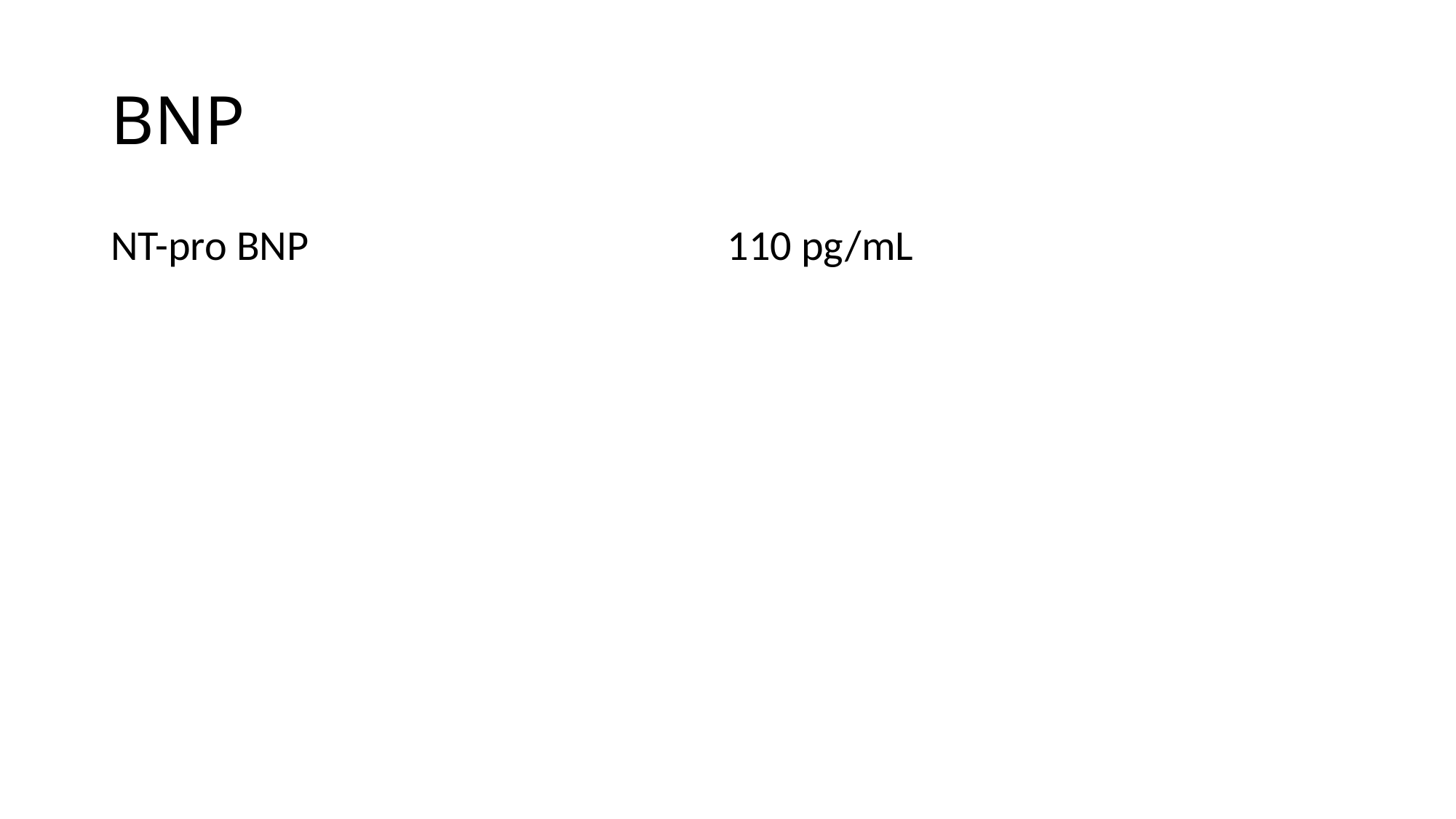

# BNP
NT-pro BNP
110 pg/mL

## Slide 6
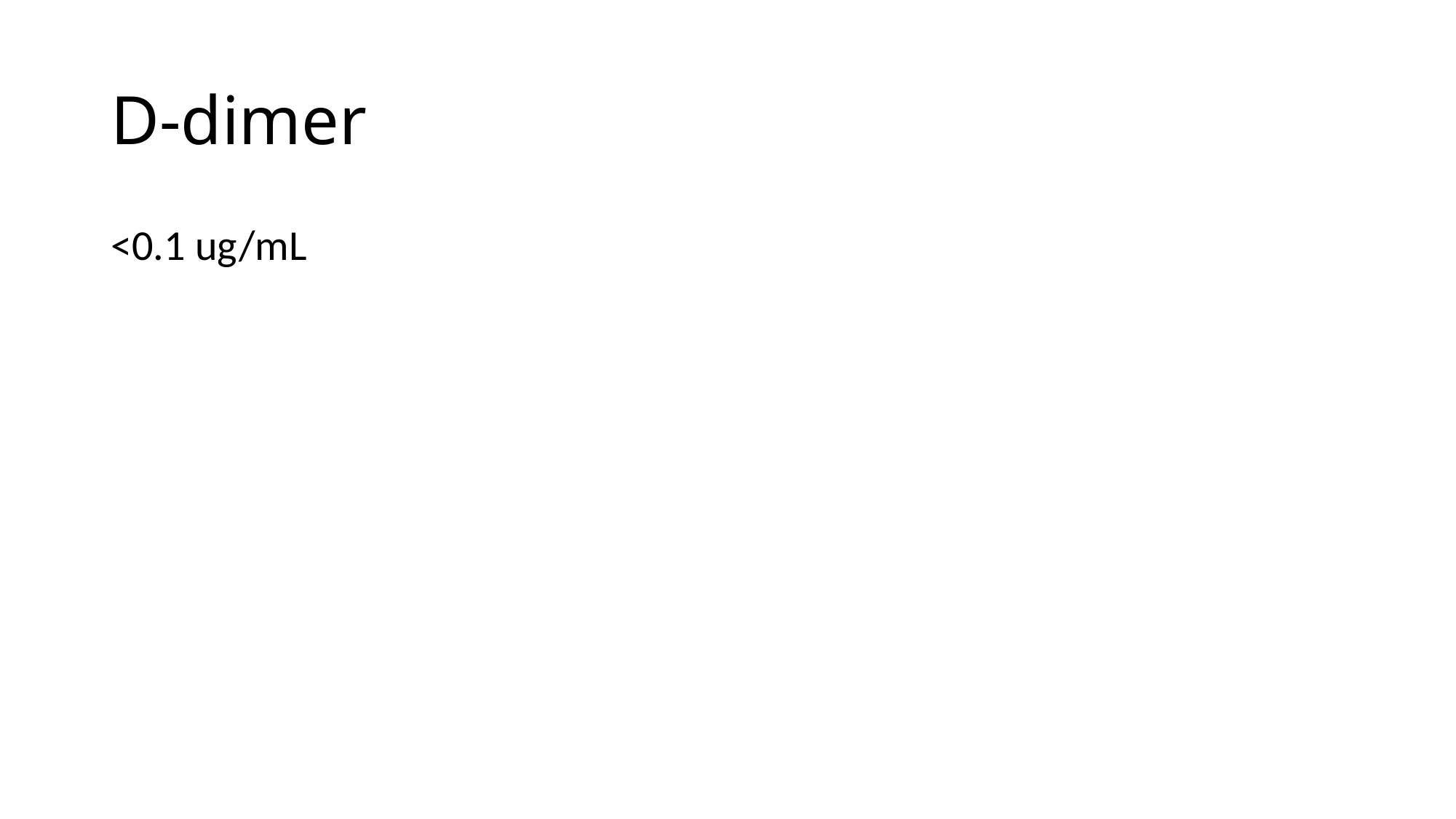

# D-dimer
<0.1 ug/mL

## Slide 7
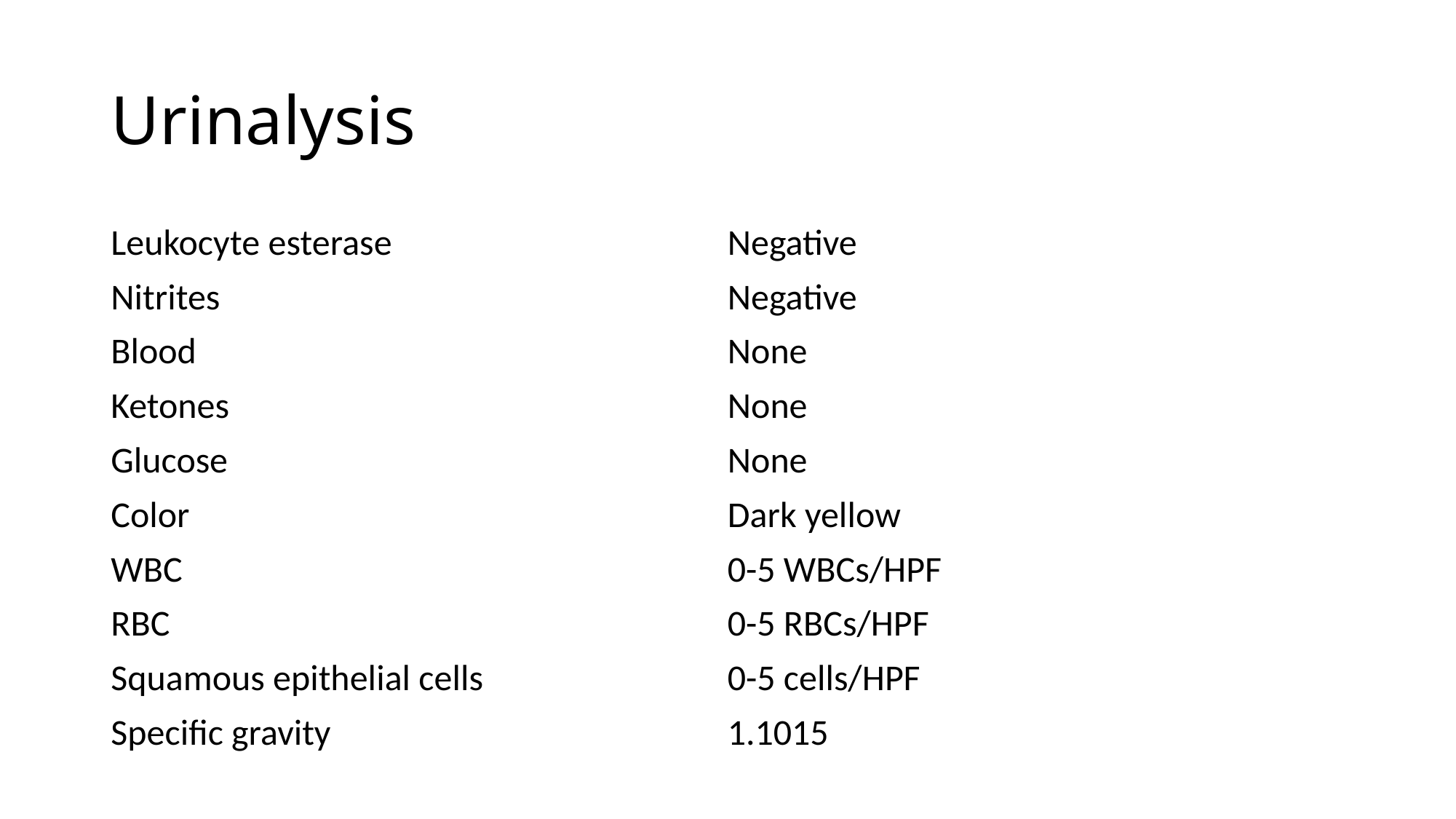

# Urinalysis
Leukocyte esterase
Nitrites
Blood
Ketones
Glucose
Color
WBC
RBC
Squamous epithelial cells
Specific gravity
Negative
Negative
None
None
None
Dark yellow
0-5 WBCs/HPF
0-5 RBCs/HPF
0-5 cells/HPF
1.1015

## Slide 8
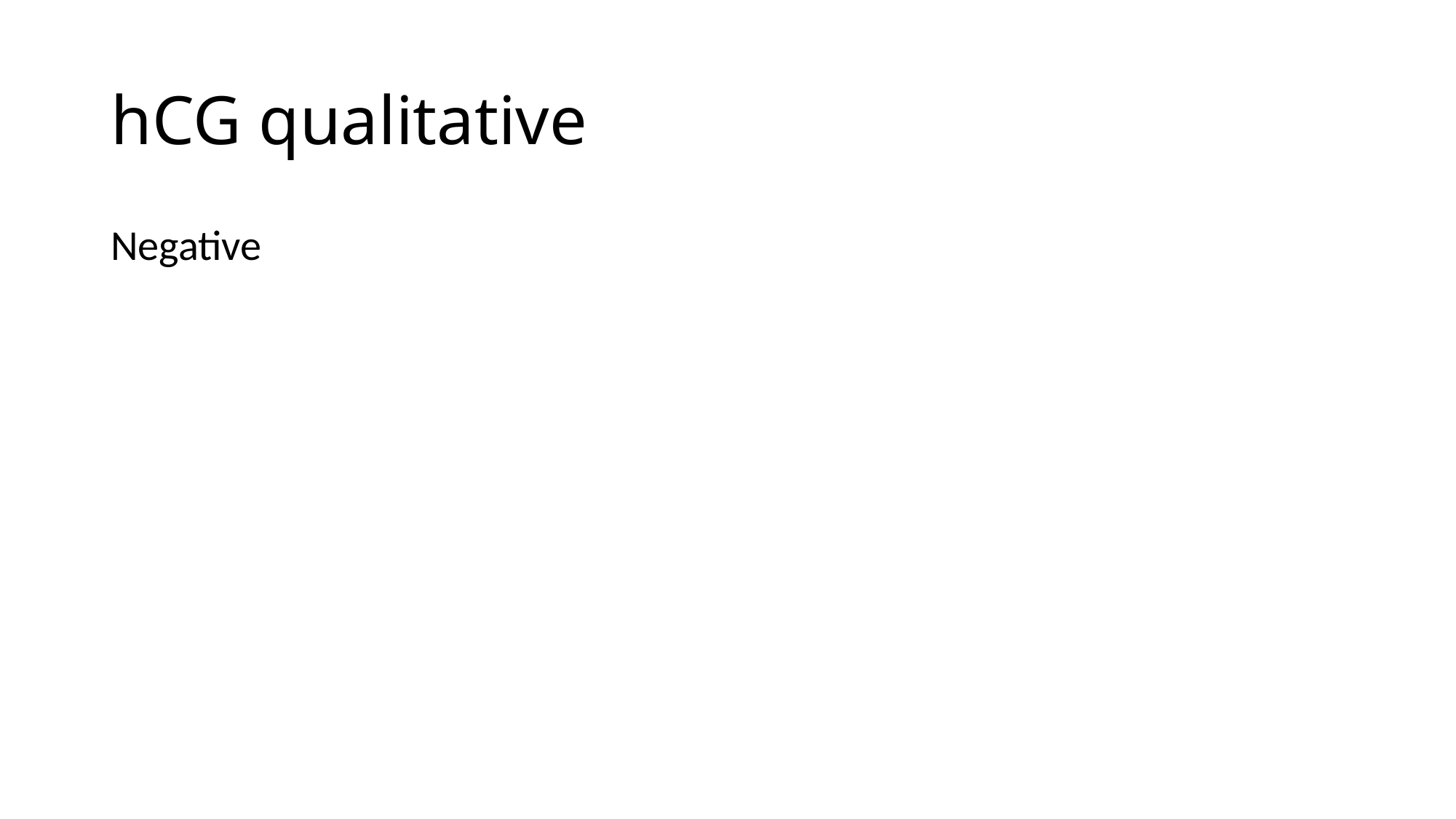

# hCG qualitative
Negative

## Slide 9
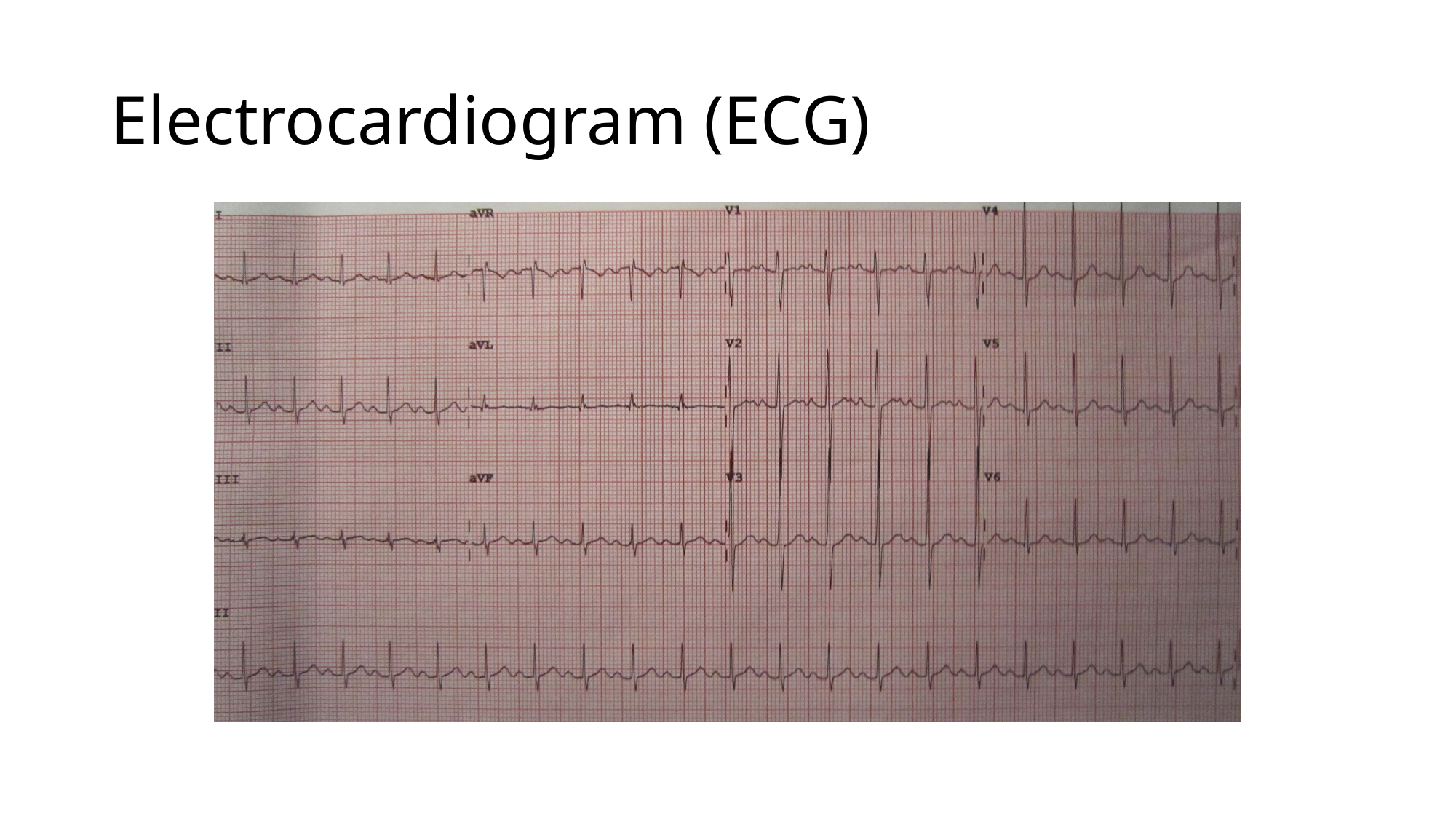

# Electrocardiogram (ECG)

## Slide 10
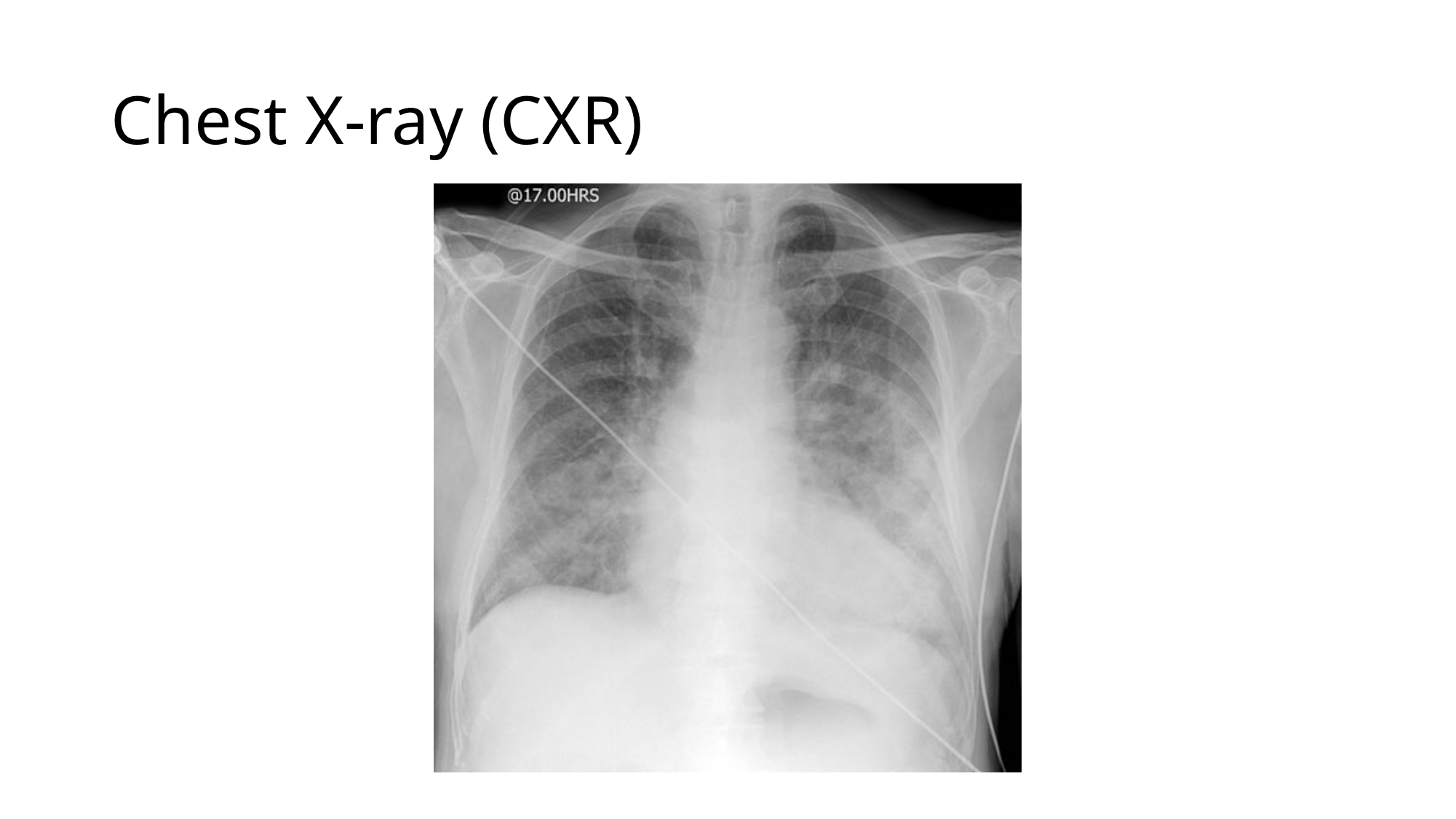

# Chest X-ray (CXR)

## Slide 11
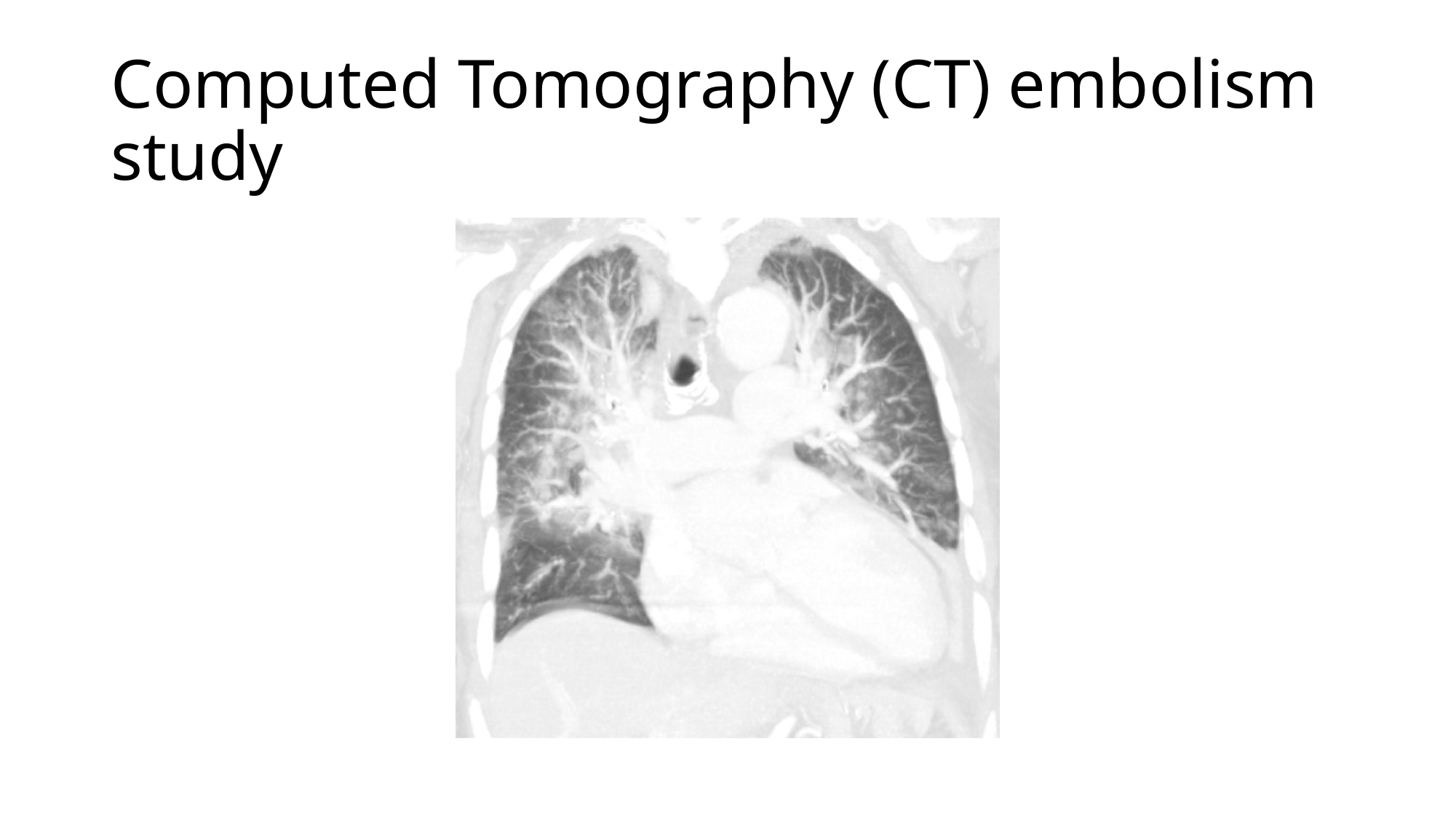

# Computed Tomography (CT) embolism study
